# CD4 Measurements in Patients with HIV: Are They Feasible for Poor Settings?

**DOI:** 10.1371/journal.pmed.0020214

**Published:** 2005-07-19

**Authors:** Zvi Bentwich

## Abstract

The lack of facilities to measure CD4 counts in poor countries impedes instituting rational and effective antiretroviral therapy in these countries. Can a new microchip counting technique help to solve the problem?

Measurement of peripheral blood CD4 T lymphocytes is probably the most important laboratory assay for evaluation and monitoring of patients with HIV. The CD4 count is critical for determining the clinical stage of HIV infection, for deciding when to start antiretroviral therapy (ART), for evaluating the efficacy of treatment, and for changing the medications when necessary. Most HIV treatment decisions are therefore based upon the CD4 count [1–3].

## Flow Cytometry

The most common technique for measuring CD4 counts in developed country settings is flow cytometry. Flow cytometers use lasers to excite fluorescent antibody probes specific for various cell surface markers, such as CD3, CD4, and CD8, which distinguish one type of lymphocyte from another.

As Rodriguez et al. point out in their study in this issue of *PLoS Medicine* [4], the cost of a flow cytometer ranges from $30,000 to $150,000, and the reagents needed for determining the lymphocyte surface markers by this method are very costly. In addition, use of flow cytometry requires technical and operational expertise as well as a reliable electricity source. Considering all these factors together, it is no surprise that CD4 measurements cannot be widely applied in developing world settings.

## Why CD4 Counts Matter in Developing Countries

This grim reality—the lack of facilities to measure CD4 counts in poor countries—stands in sharp contrast to the urgent need for instituting rational and effective ART in these countries. The absence of tools to measure CD4 counts clearly jeopardizes the success of the recently launched global campaigns to fight AIDS, such as those of the World Health Organization and the Global Fund to Fight AIDS, Tuberculosis, and Malaria. These campaigns aim to distribute ART to millions of people with HIV, mostly living in developing countries. Regretfully, it is highly likely that these major efforts will fail, unless improved and widely used means for counting CD4 cells become available and can be applied where they are most needed. Since at least 35 million people are infected with HIV, and several million of them are in need of urgent lifesaving ART, the issue of CD4 monitoring has become a crucial one.

Rodriguez et al. point out that several efforts have been made to develop alternative, affordable CD4 counting methods for resource-poor settings [4]. These include improved flow cytometric approaches and microbead capture/separation of CD4 cells followed by manual cell counting [5–8]. Also, new single-purpose flow cytometers have been designed that perform the test at a much lower price. Though all these assays are indeed cheaper than regular flow cytometry, they suffer from decreased accuracy and, most importantly, they are all of low throughput.

## A New Method for Counting CD4

Rodriguez et al. describe a novel method for counting CD4 in resource-poor settings (Figure 1) [4]. The method is based on a novel microchip detection system for measuring various analytes in very small volumes. A series of chemical and immunological reactions carried out on microspheres are visualized and captured on a charge-coupled device (developed for digital camera technology). This method allows for accurate measurement of CD4, CD8, and CD4/CD8. The prototype used for demonstration of the new apparatus shows extremely good agreement with currently used flow cytometry. Most importantly, the investigators claim that the cost of each assay is much lower than that for flow cytometry.

**Figure 1:**
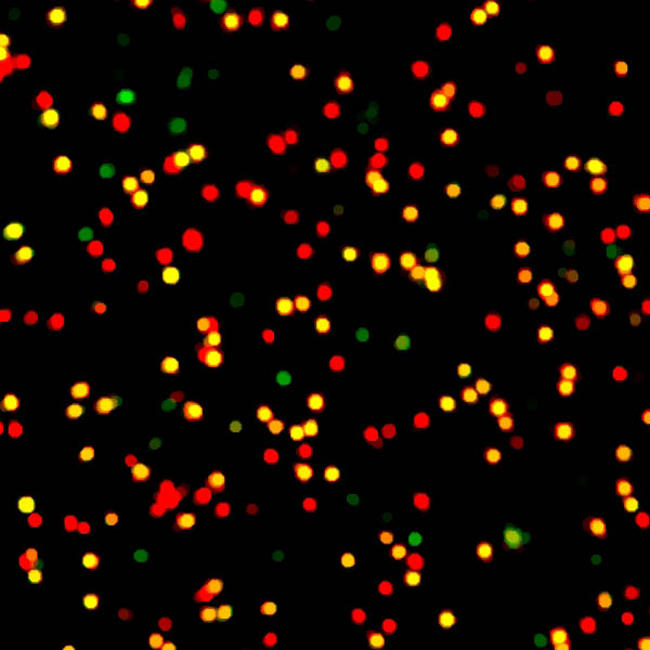
CD4 Cell Measurement Using a Prototype Microchip Counting Method This is a digital image of whole blood from a five-month-old male infant from Botswana with an absolute CD4 count of 2,098 cells/ml and a CD4 percentage of T cells of 0.39, obtained using a prototype method for low-cost CD4 count monitoring. CD4^+^ T cells are yellow. Also visualized are monocytes (green) and CD8^+^ T cells (red). (Photo courtesy of the authors of [8])

There are, however, a number of unresolved issues in this study that need further clarification before the assay can meet the expectations for becoming a widely used tool in resource-poor settings. Firstly, the study was performed with a prototype apparatus, tailored to meet the requirements of the study, but not yet representing a commercially established and viable production line. Secondly, though the authors state that the price of each CD4 determination will become much cheaper, it is not clear how much each assay will cost in the end, and whether the final cost is realistic in the context of developing countries. It is clear, though, that the actual price of the assay will change once it is widely and consistently used on a large scale. Thirdly, although a few children were tested (six infants in total), the results in this small group remain questionable, and therefore the application of the test to pediatric populations needs further testing. It may well be that application to pediatric patients will require an improved apparatus or improved handling.

## Conclusion

Despite these reservations, the authors of this study should be commended for addressing an extremely important issue and developing this novel approach for counting CD4 in patients with HIV. Their study may lead to further development of such an apparatus, which is sorely needed for the global fight against AIDS. Such efforts will hopefully be noticed by public funding agencies, leading to the improvement of tools for measuring CD4 counts.

## Abbreviation

ART: antiretroviral therapy
